# A theater production to promote a smoke-free life to secondary students

**DOI:** 10.1017/S1463423619000367

**Published:** 2019-07-01

**Authors:** Alice Yuen Loke, Yim-wah Mak, Cynthia ST Wu, Yuen-ting Wong

**Affiliations:** School of Nursing, The Hong Kong Polytechnic University, Hong Kong, China SAR

**Keywords:** peer-led program, smoke-free life, student health ambassadors, theater production

## Abstract

**Background:**

Peer-led school-based anti-smoking programs have been shown to affect the smoking behaviors of students. The aim of this study was to examine the effectiveness of a school-based peer-led live theater production advocating a smoke-free life.

**Methods:**

This is a cross-section design study. Students from the drama club were recruited as School Health Ambassadors (SHAs). The SHAs were to involve in a theater production in advocating a smoke-free life, and were provided a health education workshop from the project team on facts relating to smoking and smoke-free life. All the students in the school were to watch the theater production as school peer audience members (SPAs). Comparison will be made between the two groups of students in their attitude and decision towards living a smoke-free life after being involved in the theater production or in watching the drama.

**Results:**

A total of 409 students, 21 SHAs, and 388 SPAs were included in the project. Both the SHAs and the SPAs reported confidently about their ability to resist offers or temptation to smoke, and were determined to live a smoke-free life and refrain from smoking the first cigarette.

**Conclusions:**

A peer-led theater production advocating a smoke-free life shows some effects on students’ attitude and decision to resist offers and the temptation to smoke, and to come to the decision to live a smoke-free life and refrain from smoking the first cigarette.

## Introduction

Smoking habits are likely to be initiated during adolescence. Smoking is a progressive process, leading from initiation to occasional experimentation, and then to regular smoking (Barman *et al*., 2004). There is evidence showing that the percentage of adult regular smokers took up smoking when they were teenagers is as high as 82% (Kobus, 2003; Krauth, 2005). In Hong Kong, over half (64.8%) of daily cigarette smokers started their smoking habit between the ages of 10 and 19 (Census Statistics Department, 2008).

Public actions of tobacco control have been taken in Hong Kong, including banning smoking in public places such as restaurants, and placing anti-smoking messages on bill boards in the mass transportation system and in the mass media. Given that various efforts to help adult smokers quit have not had satisfactory results, strategies should target adolescents to prevent them from starting smoking. Adolescents usually do not expect to become addicted, and are therefore often inclined to experiment with cigarettes (Rugkasa *et al.*, 2001). Strategies should be targeted at keeping adolescents away from taking their first cigarette, establishing the intention to not smoke, and interrupting the progression from experimental smoking to addiction. Among students, reducing the rate of the intention to smoke and to experiment with smoking are powerful ways to reduce the prevalence of adult smoking in the future (Sowdon and Arblaster, 1999).

A school-based health promotion program can reach adolescents during the developmental stage when a healthy smoke-free lifestyle is established (National Assembly for Wales, 2001). Smoking prevention programs in schools targeted at preventing adolescents from initiating smoking have a potentially beneficial effect on the students over their lifetime and reduce the prevalence of adult smoking (Jit *et al*., 2010). In fact, the social network of the peer group has been identified as a significant factor influencing lifestyle choices and decisions regarding experimenting with risky behaviors (Loke and Mak, 2013). It is speculated that because peers share similar characteristics and experiences, the health messages that they deliver may be more readily persuasive than those from teachers, health educators, or parents (Milburn, 1995; Petty *et al.*, 2014). Thus, a peer-led smoking prevention program, conveying the undesirability of smoking, should produce promising effects.

A study also showed that having peers who are smokers is the most important factor in the taking up of smoking among adolescents (Loke *et al*., 2016). Changing the “norm” of smoking among students and a school-based peer-led smoking prevention program is an enriching opportunity for the peers themselves. In a project on “promoting smoke-free homes (SFH)” targeting school-aged children in schools, students pledged to promote a smoke-free environment to their families and friends, indicated that students could be effective health ambassadors for their friends in normalizing smoke-free living.

### A school-based peer-led theater production approach advocating a smoke-free life

Adolescents are highly impressionable; depictions of smoking in movies send a powerful message to adolescent viewers and indirectly influence their attitudes and smoking behavior (Sargent *et al.*, 2001). Theater production has been used as an effective educational tool for reducing risk-taking behaviors among adolescents (Bell-Ellison *et al*., 2009). Of particular interest is the report that mounting live theater productions is a potentially powerful smoking prevention strategy (Sargent *et al.*, 2001).

Live theater, such as a drama/a play on stage, is a suitable medium for communicating preventive messages to young audiences, and can complement traditional strategies for dealing with risky behaviors and peer pressure. Theater production is also an approach adopted by the Council on Smoking and Health to promote smoke-free message among students in primary schools (COSH, 1995). It is postulated that exposure to “scenarios” that normalize smoke-free behavior, suggestions of the social undesirability of smoking, and demonstrations of skills on how to refuse to smoke may have a positive effect on adolescents. This approach has neither been utilized systematically among students in Hong Kong nor have the effects been examined.

The element of live theater productions in schools will attract the attention of students and send a powerful message to those involved in the productions and to their peer audiences. Students who are at risk of experimenting with cigarettes can then be reached, to prevent the early uptake of cigarettes, reduce the overall rates of smoking adolescents developing into adult smokers, and reduce the overall smoking population in Hong Kong.

### Project aims and objectives

The aim of this project was to examine and compare the effectiveness of a school-based peer-led live theater production advocating a smoke-free life. The specific objectives of this study were to examine: (1) the smoking habits and smoking environment of secondary students (2) their confidence in resisting offers or the temptation to smoke, and (3) attitudes and decisions towards living a smoke-free life, after being involved in production or watching a theater production advocating a smoke-free life.

## Project method

### Project design and setting

This was a health promotion project of a school-based peer-led live theater production advocating a smoke-free life. The study was conducted in a co-educational secondary school in Hong Kong from July 2014 to April 2015.

### Participants

The student members in the drama club of the school were recruited, constituted the theater production team, and were recruited as School Health Ambassadors (SHAs). There were a total of 21 students in the drama club. The SHAs received health education workshop from the members of the project team regarding the facts and impacts of smoking and smoke-free life. All students in the secondary school were to watch the theater production produced by the SHAs as school peer audiences (SPAs). No health education was given by the project team directly to the SPAs.

All students, SHAs and SPAs, were included regardless of their smoking status. This decision was made so as to overcome a shortcoming commonly identified in peer-led smoking prevention studies, that there has been little involvement from smoking students (Audrey *et al.*, 2006).

### Development of the intervention: playwright and theater production

A specially designed health education workshop was conducted targeting all recruited SHAs, the drama teacher, and the drama director. It was conducted by a member of the research team, who has experience in conducting training sessions for young people on smoking counseling and the promotion of SFH. The health education workshop emphasized subjects such as the normalization of a smoke-free life, the benefits of maintaining a smoke-free environment, the prevalence of smoking among adolescents in Hong Kong, social influence on smoking behaviors, strategies to refrain from experimenting with smoking, resisting peer pressure, and skills on how to refuse offers of cigarettes.

The members of the research team, the drama teacher, and the director worked together to facilitate the discussion among the SHAs on issues that concerned them most about smoking, the consequences of smoking, the reasons for experimenting with smoking, and benefits of quitting smoking. The principles of helping people with empathy and concern, effective communication skills, and how to appreciate the effects and benefits of peer-led live theater productions were shared.

The SHAs, with the guidance of their drama teacher, took up roles as playwrights, directors, actors/actresses, and backstage crew. They worked together to write and create a theater production advocating a smoke-free life. The research team/director held regular meetings with the production team to answer all queries, to monitor the progress, and to control the quality of the production. The plots and script were scrutinized by the drama teacher/director and the members of the research team to ensure that information contained in the drama relating to active and passive smoking, and strategies for living a smoke-free life were accurate and appropriate, and aligned with the objectives of the intervention.

The theater production, which was 45 min long, was finally performed in April 2015 in the school auditorium as a health promotion activity, with all students of the school (except those who were absent on that day) attending as SPAs.

### Instrument

A questionnaire to be completed by the SHAs and the SPAs was specially designed for this study. The questionnaire consisted of four sections, with the first section soliciting the students’ demographic data, such as age, gender, and family members. The second section collected information on self-reported smoking status, the smoking habits of parents, siblings, and peers, as well as their perception of the smoking norms in their home and of the school’s social environment. The third section consisted of items on the students’ self-efficacy in resisting offers or different scenarios tempting them to smoke, and their smoking intention.

All items were based on existing pre-validated questions and have been used among Chinese adolescents in previous studies (Abdullah *et al*., 2005; Loke and Wong, 2010; Loke and Mak, 2015). Self-reported smoking status and self-efficacy to resist offer of cigarettes have been shown to be reliable among adolescents (Perry *et al*., 1999) and among Chinese (Mak *et al*., 2005).

As it is the intention of this project to examine the impacts of the theater production to promote smoke-free life, the last section contained 14 questions pertaining to the characters and plots in the drama produced by the production team. These items were developed to examine if the message in the drama came across to the students. These questions focused on the actions of the characters in the drama, their intention to not smoke, the norms that they expressed, the influence of peers, their resistance of experimental smoking, and refusal of offers of cigarettes. The students were asked to indicate what they had learned from the characters after being involved in the production or as part of the audience. They were asked to indicate their agreement with the attitude and actions related to living a smoke-free life by using a 4-point Likert scale (strongly agree, agree, disagree, and strongly disagree).

### Validity and reliability

The items in questionnaire were developed based on pre-validated questions (Abdullah *et al*., 2005; Loke and Wong, 2010; Loke and Mak, 2015). The questionnaire was piloted among 11 secondary students in March 2015 to examine the clarity of the questions. Minor changes were made to the wordings according to the comments received from the students who completed the pilot test. The test-retest reliability was established, with *r* = 0.70, 0.60, 0.63, and 0.76 for smoking status (4 items), smoking attitude, accessibility and environment (8 items), and self-efficacy (12 items) in resisting offers or the temptation to smoke, and attitude towards a smoke-free life (4 items).

### Ethical considerations

Ethical approval for the study was obtained from the Human Subject Ethics Subcommittee of the Hong Kong Polytechnic University. The school invited to participate in the study was given a detailed explanation of the study protocol. The authorities of the participating school returned a *pro forma* document indicating their willingness to take part in the study. A meeting was arranged, and a detailed plan and explanation of the project was given to the drama teacher who was involved. Parents were informed of the activities and have the right to return a refusal form. Both school principal and parents were informed that the results of the study might be published for the purpose of scholarly output, and they were assured that the confidentiality of the students will be maintained. Data were collected anonymously, and the identities of the SHAS and SPAs will not be revealed. The students were told that they had the right to not return the questionnaire that was distributed without suffering any penalty.

## Data collection and analysis

Self-administered questionnaires were distributed to both the SHAs and SPAs after the performance of the theater production. The school principal and the drama teacher provided students with a clear explanation of the study. All of the teachers helped to distribute the questionnaires to the students in the auditorium of the school immediately after the theater production, and collected them when they were completed. The questionnaires for SHAs were printed in color papers to differentiate from the questionnaires for SPAs by the drama teacher. Students whose parents did not return the refusal form to take part in the study, and the students who completed and returned the questionnaires to the teachers were considered to have consented to participate in the study.

All of the collected data were entered and analyzed using SPSS Version 21. Descriptive statistics were used to describe the students’ demographics, family characteristics, and perception of the following items relating to living a smoke-free life: smoking habits, accessibility and attitude towards smoking, and awareness of smoking policies in the students’ social environment; self-perceived confidence in resisting offers or the temptation to smoke; and the students’ attitude and decision to live a smoke-free life after being involved/watching the theater production. A Chi-square test was used to determine any differences in proportion between SHAs and SPAs on all of the related items. Fisher’s Exact Test was used for cells with count of less than five. The level of statistical significance was set at *P* < 0.05.

## Results

A total of 459 questionnaires were collected from the SPAs, and 21 from the SHAs who took part in the theater production on promoting a smoke-free life. Among the 459 questionnaires collected from the SPAs, 71 were incomplete; as a result, only 388 questionnaires (84.5%) were included for analysis in this report.

### Demographics and family characteristics

The total of 409 secondary school students, 21 SHAs and 388 SPAs, consisted of 51.1% male and 47.2 % female students. The students had a mean age of 14.8 years (Table 1). Both junior year (57.0%) and senior year students (42.3%) were included. Over 80% of the students were living with their father/mother and about 83.6% had siblings. There were no significant differences in student and family characteristics between SHAs and SPAs, including in the educational attainment and employment of their parents.

**Table 1.** Demographics and family characteristics of secondary school students (School Health Ambassadors & School Peer Audiences) (*N* = 409)

	Total (*N* = 409)	School health ambassadors (*n* = 21)	School peer audiences (*n* = 388)	
Characteristics	*N* (%)	*n* (%)	*n* (%)	*P* ^a^
*Gender* ^b^				
Male	209 (51.1)	12 (57.2)	197 (50.8)	0.63
Female	193 (47.2)	9 (42.9)	184 (47.4)	
*Age* (mean ± sd)^b^	14.83 ± 1.6	15.05 ± 1.6	14.82 ± 1.6	0.52
*Education Level* ^b^				
F.1-F.3 (junior years)	233 (57.0)	11 (52.4)	222 (57.2)	0.63
F.4-F.6 (senior years)	173 (42.3)	10 (47.6)	163 (42.0)	
*Family members living with*				
Father	330 (80.7)	17 (81.0)	313 (80.7)	1.00
Mother	345 (84.4)	17 (81.0)	328 (84.5)	0.76
Siblings	292 (71.4)	14 (66.7)	278 (71.6)	0.62
Grandparents/ relatives	21 (5.1)	1 (4.8)	20 (5.2)	1.00
*Have sibling(s)*	342 (83.6)	15 (71.4)	327 (84.3)	0.12
*Educational attainment of the father* ^c^				
Primary school or below	73 (17.8)	7 (33.3)	66 (17.0)	0.13
Secondary school	252 (61.6)	10 (47.6)	242 (62.4)	
Tertiary school	55 (13.4)	2 (9.5)	53 (13.7)	
*Educational attainment of the mother* ^c^				
Primary school or below	92 (24.0)	5 (23.8)	87 (22.4)	0.93
Secondary school	246 (64.1)	14(66.7)	232 (59.8)	
Tertiary school	46 (12.0)	2 (9.5)	44 (11.3)	
*Occupation of the father* ^c^				
Employed	356 (87.0)	19 (90.5)	337 (86.9)	0.57
Unemployed	15 (3.7)	1 (4.8)	14 (3.6)	
*Occupation of the mother* ^c^				
Homemaker	181 (44.3)	8 (38.1)	173 (46.8)	0.63
Employed	199 (48.7)	11 (52.4)	188 (51.1)	
Unemployed	9 (2.2)	1 (4.8)	8 (2.2)	

a Statistical tests included the Chi-square test and the Mann–Whitney U test. For cells with count of less than five, Fisher’s Exact Test was adopted.

b Missing data constituted less than 5% (range 0.7–4.9%).

c Missing data constituted more than 5% (range 6.4–9.3%).

### Smoking habits, attitude, accessibility, and social environment related to smoking

The smoking habits, accessibility and attitude towards smoking, and awareness of smoking policies of SHAs and SPAs in the students’ social environment are shown in Table 2.

**Table 2.** Smoking habits and environment (*N* = 409)

	Total (*N* = 409)	School health ambassadors (*n* = 21)	School peer audiences (*n* = 388)	
	*N* (%)	*n* (%)	*n* (%)	*P* ^a^
*Smoking habits*				
Smoking status of the student^d^				
Never tried smoking	370 (90.5)	19 (90.5)	351 (90.5)	0.68
Ever tried smoking	32 (7.8)	2 (9.5)	30 (7.7)	
Number of cigarettes smoked per day^b^ (median range)	(*n* = 32) 4.5 (1.8-20.0)	(*n* = 2) N/A	(*n* = 30) 4.5 (1.8-20.0)	N/A
Smoking status of the father^d^				
Non-smoker	173(42.3)	11 (52.4)	162 (41.8)	0.67
Former smoker	67 (16.4)	3 (14.3)	64 (16.5)	
Regular smoker	162 (39.6)	7 (33.3)	155 (39.9)	
Smoking status of the mother				
Non-smoker	353 (86.3)	18 (85.7)	335 (86.3)	0.98
Former smoker	16 (3.9)	1 (4.8)	15 (3.9)	
Regular smoker	40 (9.8)	2 (9.5)	38 (9.8)	
Smoking status of the sibling(s) ^c^(*n* = 342)		(*n* = 15)	(*n* = 327)	
Non-smoker	307 (89.8)	10 (66.7)	297 (90.8)	0.003**
Smoker	35 (10.2)	5 (33.3)	30 (9.2)	
Smoking habit of friends^d^				
Do not have friends who smoke	299 (73.1)	16 (76.2)	283 (72.9)	0.69
Have one friend who smokes	13 (3.2)	0 (0.0)	13 (3.4)	
Have two or more friends who smoke	90 (22.0)	5 (23.8)	85 (21.9)	
*Parents and friends’ attitude towards smoking*				
My parents oppose smoking^d^	344 (84.1)	19 (90.5)	325 (83.8)	0.75
My friends oppose smoking^d^	317 (77.5)	11 (52.4)	306 (78.9)	0.003**
*Accessibility and home environment*				
Cigarettes can easily be found in my home^d^	105 (26.7)	8 (38.1)	97 (25.0)	0.19
My parents will notice if I try smoking^d^	319 (78.0)	18 (85.7)	301 (77.6)	0.59
Smoking policy at home				
Smoking is not allowed in my house	214 (52.3)	12 (57.1)	202 (52.1)	0.40
Smoking is allowed when visitors smoke	52 (12.7)	4 (19.0)	48 (12.4)	
Smoking is allowed in any situation	70 (17.1)	4 (19.0)	66 (17.0)	
Not aware of any policy at home	73 (17.8)	1 (4.8)	72 (18.6)	
*Awareness of non-smoking policy at school*				
Have always seen other students smoking near school	178 (43.5)	9 (42.9)	169 (43.6)	0.95
My school has a policy on restricting smoking	347 (84.8)	19 (90.5)	328 (84.5)	0.75
Violators of the school’s non-smoking policy will be subjected to punishment	372 (91.0)	21 (100.0)	351 (90.5)	0.24

a Statistical tests included the Chi-square test and Mann–Whitney U test. For cells with count of less than five, Fisher’s Exact Test was adopted to obtain the *P* value.

b Among those who had ever tried smoking only.

c Among those with siblings only.

d Missing data constituted less than 5% (range 0.4–1.7%).

** *P* ≤ 0.01.

In general, the majority of the students had never tried to smoke (*n* = 370, 90.5%), where 32 (7.8%) of them admitted to have ever tried smoking. Most of them (86.3%) had non-smoking mothers, 89.8% had non-smoking siblings (among those with siblings), and 73% had no friends who smoked. However, over half of the fathers of these students were former or regular smokers (56.0%). Among those with siblings, higher proportion of SHAs than SPAs had siblings who smoke (33.3% versus 9.2%, *P* = 0.003). Most parents and friends of the students opposed smoking (84.1% and 77.5%, respectively). SHAs were less likely than SPAs to perceive that their friends oppose smoking (52.4% versus 78.9%, *P* = 0.003).

About 26.7% of students reported that they can access cigarettes at home, but 78% said that their parents would notice if they were to smoke. Over half of the students reported that smoking is not allowed in their home (52.3%). As many as 43.5% of the students have witnessed their peers smoking near the school, although 84.8% were aware of the policy restricting smoking in school, and 91% understood that those violating the non-smoking policy at school would be subject to punishment.

### Self-efficacy in resisting offers or temptations to smoke

The self-perceived efficacy of SHAs and SPAs to resist offers or temptations to smoke is shown in Table 3. The percentages indicate the portion of students who perceived themselves as being very confident or confident that they would be able to resist offers or temptations to smoke in different scenarios. Most of the students were confident of being able to resist smoking when walking back home (84.4%), but less were confident about being able to refrain from smoking in the future (78.7%). Overall, SHAs were more confident than SPAs about being able to resist offers or temptations to smoke in different situations, especially when they were with friends who smoke (100.0% versus 82.2%, *P* = 0.03).

**Table 3.** Students’ self-efficacy in resisting offers or temptations to smoke (*N* = 409)

	Total (*N* = 409)	School health ambassadors (*n* = 21)	School peer audiences (*n* = 388)	
	*N* (%)	*n* (%)	*n* (%)	*P* ^a^
*Very confident and confident about my ability to resist offers or temptations to smoke*				
when a friend offers me a cigarette	331 (80.9)	18 (85.7)	313 (80.7)	0.78
when I am with other smokers	332 (81.2)	20 (95.2)	312 (80.4)	0.15
when I am with a friend who smokes	340 (83.1)	21 (100.0)	319 (82.2)	0.03*
when I am upset	333 (81.4)	20 (95.2)	313 (80.7)	0.15
when I am nervous^b^	334 (81.7)	18 (85.7)	316 (81.4)	0.78
when I am worried	341 (83.4)	20 (95.2)	321 (82.7)	0.22
when I go shopping with friends^b^	339 (82.9)	20 (95.2)	319 (82.2)	0.23
when I am watching TV^b^	340 (83.1)	19 (90.5)	321 (82.7)	0.55
when I am doing homework^b^	339 (82.9)	19 (90.5)	320 (82.5)	0.55
when I am walking back home^b^	345 (84.4)	20 (95.2)	325 (83.8)	0.22
I definitely will not smoke within the next year^b^	329 (80.4)	20 (95.2)	309 (79.6)	0.09
I definitely will not smoke in the future^b^	322 (78.7)	20 (95.2)	302 (77.8)	0.09

a For cells with count of less than five, Fisher’s Exact Test was adopted to obtain the *P* value.

b Missing data constituted less than 5% (range 0.2–1.2%).

* *P* ≤ 0.05.

### Attitude and decision to live a smoke-free life after being involved in/watching the theater production

The students’ attitude towards messages about living a smoke-free life according to the plot or characters in the drama and the pledge to live a smoke-free life after being involved in or watching the theater production are shown in Table 4. The percentages indicate the portion of students who strongly agree with the plot/characters in the drama or with the pledge to live a smoke-free life.

**Table 4.** Attitude and decision towards living a smoke-free life after being involved in/watching the theatre production (*N* = 409)

	Total (*N* = 409)	School health ambassadors (*n* = 21)	School peer audiences (*n* = 388)	
	*N* (%)	*n* (%)	*n* (%)	*P* ^a^
*Strongly agree that the plot/ characters in the drama taught:*				
More understanding of a smoke-free life	183 (44.7)	13 (61.9)	170 (43.8)	0.10
More awareness of and attention to a smoke-free life	160 (39.1)	13 (61.9)	147 (37.9)	0.03*
That the smell of cigarettes can stay on me and my hair	179 (43.8)	14 (66.7)	165 (42.5)	0.03*
That smoking can be addictive and ruin my life	192 (46.9)	17 (81.0)	175 (45.1)	0.001**
Smoking can cause premature wrinkles, yellow, teeth and halitosis^d^	188 (46.0)	19 (90.5)	169 (43.6)	<0.001**
That smoking can hurt our family members^d^	165 (40.3)	11 (52.4)	154 (39.7)	0.25
That smoking hurts nearby people^d^	176 (43.0)	14 (66.7)	162 (41.8)	0.03*
That smoking can cause pollution to the environment^d^	181 (44.3)	16 (76.2)	165 (42.5)	0.003**
That smoking can have damaging effects on health and can cause early death^d^	190 (46.5)	16 (76.2)	174 (44.8)	0.005**
That smoking is not an effective method for losing weight^d^	188 (46.0)	16 (76.2)	172 (44.3)	0.002**
That I can learn to persuade/advise family members or friends to stay away from cigarettes^d^	181 (44.3)	15 (71.4)	166 (42.8)	0.005**
That young children are more likely to smoke if their parents smoke^d^	179 (43.8)	13 (61.9)	166 (42.8)	0.05*
*Among those who smoke:*	(*n* = 32)	(*n* = 2)	(*n* = 30)	
I would quit smoking immediately ^b^	7 (21.9)	1 (50.0)	6 (20.0)	0.40
I would not smoke at home ^b^	10 (31.2)	1 (50.0)	9 (30.0)	0.53
*Among those with parents who smoked:*	(*n* = 256)	(*n* = 14)	(*n* = 242)	
I would ask them to quit smoking immediately ^c^	131 (51.2)	8 (57.1)	123 (50.8)	0.65
I would ask them not to smoke at home ^c^	131 (51.2)	9 (64.3)	122 (47.7)	0.31
*Strongly agree to pledge to live a smoke-free life:*				
I pledge that I will never smoke^d^	263 (64.3)	16 (76.2)	247 (63.7)	0.27
I have reduced my intention to ever smoke^d^	181 (44.3)	18 (85.7)	163 (42.0)	<0.001**
I have increased my ability to refuse the temptation tosmoke^d^	180 (44.0)	16 (76.2)	164 (42.3)	0.003**
I am determined to refrain from smoking and to lead a smoke-free life	201 (49.1)	17 (81.0)	184 (47.4)	0.003**
It is easy to smoke but difficult to quit, therefore, I shall refrain from picking up the first cigarette^d^	195 (47.7)	17 (81.0)	178 (45.9)	0.003**
I will promote messages of a smoke-free life to my family, relatives, or friends^d^	170 (41.6)	13 (61.9)	157 (40.5)	0.05*

a For cells with count of less than five, Fisher’s Exact Test was adopted to obtain the *P* value.

b Among those who have ever tried smoking.

c Among those with smoking families.

d Missing data constituted less than 5% (range 0.2–1.5%).

* *P* ≤ 0.05.

** *P* ≤ 0.01.

Generally, SHAs agreed more with the messages brought from the drama than SPAs, perceiving that the drama could increase their awareness of and attention to the importance of living a smoke-free life (61.9% versus 37.9%, *P* = 0.03), reduce their intention to ever smoke (85.7% versus 42.0%, *P* < 0.001), and increase their ability to resist the temptation to smoke (76.2% versus 42.3%, *P =* 0.003). There were statistically significant differences between SHAs and SPAs in their agreement with the plot, including with items such as “the smell of cigarettes can stay on my hair and me” (66.7% versus 42.5%, *P* = 0.03), “smoking can be addictive and ruin my life” (81.0% versus 45.1%, *P* = 0.001), “smoking can cause premature wrinkling, yellow teeth, and halitosis” (90.5% versus 43.6%, *P* < 0.001), “smoking hurts people nearby” (66.7% versus 41.8%, *P* = 0.03), “smoking can cause pollution to the environment” (76.2% versus 42.5%, *P* = 0.003), “smoking can have damaging effects on health and can cause early death” (76.2% versus 44.8%, *P =* 0.005), “smoking is not an effective method of losing weight” (76.2% versus 44.3%. *P* = 0.002), “learn to persuade/advise family members or friends to stay away from cigarettes” (71.4% versus 42.8%, *P* = 0.005), and “young children are more likely to smoke if their parents smoke” (61.9% versus 42.8%, *P* = 0.05).

A higher proportion of SHAs than SPAs were determined to pledge to living a smoke-free life, including refraining from smoking and leading a smoke-free life (81.0% versus 47.4%, *P* = 0.003), refraining from picking up the first cigarette due to the fact that it is easy to smoke but difficult to quit (81.0% versus 45.9%, *P* = 0.003), and promoting messages of a smoke-free life to family, relatives, or friends (61.9% versus 40.5%, *P* = 0.05). Among students who had ever tried to smoke, their willingness to quit smoking immediately (21.9%) and not to smoke at home (31.2%) was relatively low. However, of the students from smoking families, slightly more than half were determined to ask their parents to quit smoking immediately (51.2%) and not to smoke at home (51.2%).

## Discussion

### Characteristics and generalizability of the study sample

The whole student population in the secondary school were involved in the study. The school is in a district populated with mostly middle-class families, with around 75% of the parents of the students having attained a secondary school level of education and above, compared with 67% of the population aged 15 and over in Hong Kong (Census and Statistics, 2016b). The unemployment rate of the parents was similar to that reported by the Hong Kong Census and Statistics Department (2016a). The family characteristics of the students resemble the characteristics of the general adult population in Hong Kong.

### Smoking habits, attitude, accessibility, and environment

The students in this project were exposed to smoking within their families and near school. Nearly 40% of the fathers of the students were regular smokers, and one in four students can easily find cigarettes at home. Having a father who smokes is a factor that contributes to students taking up smoking (Loke and Wong, 2010). Although most of the students were aware of the smoking restriction policy of their school, nearly half of them had witnessed other students smoking near the school, which may affect the students’ perception that smoking is a norm among people of their age and thus increasing the chance that they will take up smoking (Lai *et al*., 2004; Loke and Wong, 2010). Such social environment may be conducive to the initiation of smoking among students.

### Self-efficacy in resisting offers or temptations to smoke

Self-efficacy refers to a person’s confidence in performing certain behaviors. In this study, majority of the students perceived that they had the self-efficacy to resist smoking, even when they were with a friend who smoked after the theater production. Since peer influence is a primary factor associated with adolescent smoking behavior, and since there is a higher probability that teenagers staying with smoking friends will smoke (Kobus, 2003), having the self-efficacy to resist the temptation to smoke when they were with friends who smoke would be effective at helping the SHAs to reduce their chances of experimenting with smoking.

A recent study found that secondary students considered friendliness and the ability to be entertaining to be the top characteristics of an influential peer (Loke *et al*., 2017). Influential peers were found to be instrumental in engaging at-risk students in health promotion programs, and to have the potential to help other students to resist behaviors threatening to their health. Overall, the students in this study were confident about their ability to resist offers or temptations to smoke in different situations. This is also a good indicator that they will be able to act effectively as health ambassadors in delivering the message of smoke-free living to their peers.

The SHAs, who were involved in the theater production, were significantly more likely than the SPAs to report that they had the self-efficacy to refuse the temptation to smoke. A study suggested that being involved in a theater production instead of being only a member of the audience can help students to apply the knowledge or skills that they have learned to real-world situations by using situated learning (Anderson, 2004). Through the process of being involved in writing a play and acting a role, the SHAs’ smoking-related knowledge, refusal skills, and the perspectives of smokers and non-smokers were strengthened, which helped them to decide to live a smoke-free life (Anderson, 2004). The results of this study support the view that involvement in a theater production shows some promising effects in enhancing the ability of the students to solve real-world smoking temptation problems.

### Attitude and decision to live a smoke-free life after being involved in/watching a theater production

Generally, the students agreed with and made the decision to live a smoke-free life after the theater production intervention. SHAs were more aware of the merits of a smoke-free life after their involvement as members of the production team. Elements of the theater can draw the attention of people and arouse their interest in a particular issue through the use of entertainment (Post *et al.*, 2005). The theater production drew the attention of the students to the topic of a smoke-free life, especially the SHAs who were involved in the theater production.

It was reported in a previous study that students expressed their intention not to smoke after attending an anti-smoking theater production as members of the audience (Post *et al.*, 2005). In this study, SHAs who were involved in the theater production theater were shown to be more likely to pledge to refrain from smoking and lead a smoke-free life than SPAs, who watched the production as part of the audience. The active involvement of SHAs in the theater production demonstrated that such involvement had a stronger effect on the students’ intention to smoke and decision to live a smoke-free life. Combining health messages relating to a smoke-free life with involvement in theater production reduced the skepticism arising from the perception of a disconnection between knowledge and the real-world situation (Anderson, 2004). This theater production approach increased the students’ awareness of being smoke-free as a norm, and satisfied their curiosity about the related health messages.

After the theater production intervention, higher proportion of SHAs than SPAs strongly agreed with the idea of reducing the intention to smoke and pledged to refrain from picking up their first cigarette. Since reducing the percentage of students who intend to smoke and to experiment with smoking is a powerful means of reducing the prevalence of adult smoking in the future (Sowdon and Arblaster, 1999), this theater production peer-led program is an effective method of indirectly lowering the prevalence of smoking among the future adult population.

It was reported in a previous study that peer-led anti-smoking programs are seldom successful at reaching or persuading peers who are currently smoking of the merits of quitting smoking (Audrey et al., 2006; Campbell et al., 2008). Although the number of smoking students is small in this study, 7 out of the 32 students who ever tried smoking pledged to quit smoking immediately after the theater production. This theater production intervention reached the smoking students and had some influence on them. SHAs were also more willing than SPAs to promote the message of the merits of living a smoke-free life to their families, relatives, or friends. The fact that the SHAs received education workshop before the writing of a playwright, and direct communication with drama teacher/director could have produced additional effects on the SHAs. It can be concluded that the message of living a smoke-free life can be disseminated by SHAs to their peers and ultimately reduce the prevalence of smoking among the overall population.

## Limitations of the study

This is a health promotion project using a theater production to promote the message of living a smoke-free life. This study is limited in that there is no baseline data on relevant smoking-related variables for comparison. No follow-up session was held. Long-term changes in the students’ attitude towards a smoke-free life and their self-efficacy in resisting offers to smoke cannot be evaluated.

Another limitation of the project is related to the small number of SHAs recruited, limiting the variability of the SHA group. There were also a small number of students who ever tried smoking in the school. As students who ever tried smoking are also the target population for such health promotion project, they were not excluded from the production team as SHAs (2/21, 9.5%), or as SPAs (30/388, 7.7%). It should be noted that the percentage of daily cigarette smokers of people in Hong Kong is 10.0% in 2017, with the lowest rate for persons aged 15–19 at 1.0%. (Census Statistics Department, 2018). The inclusion of these smoking students (32/409, 7.8%) represented the smokers in this age range. Lastly, only one secondary school was involved in the study, limiting the generalizability of the results.

## Conclusions

This theater production project attracted the attention of students on the merits of living a smoke-free life. Students were better able to grasp smoking-related knowledge and practical skills on refusing to smoke when these were imparted to them through a live theater production. Further studies should be conducted to evaluate the changes in the students before and after such an intervention. The involvement of more schools with theater production could further substantiate the effectiveness of such an intervention. The effects of other education interventions/ approaches, such as role play or song writing to promote smoke-free life should be explored.

## Implications

The results of this project show that a school-based peer-led program involving a live theater production can be an effective and innovative health promoting project to reduce the intention of students to smoke and to experiment with cigarettes. SHAs, who participated in an education workshop and were involved in the production, had a slightly higher self-efficacy in resisting the temptation to smoke. This project was an attempt to reach those who were smokers or were susceptible to smoking, and hopefully through peer interaction, message of living a smoke-free life and interventions can be effectively provided to young people who needed to hear these messages.

The students in this study were exposed to smoking both from their family and from the nearby school environment. As families and schools are the main sources of influence on adolescent smoking behaviors (Kobus, 2003), it is necessary for parents and school personnel to make efforts to maintain a smoke-free environment in order to minimize the notion among students that smoking is a “norm.” A smoke-free social environment among adolescents should be achieved; parents should be aware of the influence of their smoking habits on their children, and school personnel should strive to maintain a smoke-free environment in the school.
